# Bis(5-methyl-1-phenyl-1*H*-1,2,3-triazole-4-carb­oxy­lic acid) monohydrate

**DOI:** 10.1107/S1600536810026243

**Published:** 2010-07-10

**Authors:** Jin Rui Lin

**Affiliations:** a Ordered Matter Science Research Center, Southeast University, Nanjing 210096, People’s Republic of China

## Abstract

The crystal structure of the title compound, 2C_10_H_9_N_3_O_2_·H_2_O, synthesized from azido­benzene and ethyl acetyl­acetate, is stabilized by O—H⋯O and O—H⋯N hydrogen bonds.

## Related literature

The title compound was studied as part of our search for phase transition materials, see: Li *et al.* (2008 ▶); Zhang *et al.* (2009 ▶). For the preparation, see: El Khadem *et al.* (1968 ▶). For the biological activity of triazoles, see: Olesen *et al.* (2003 ▶) Tian *et al.* (2005 ▶). For a related structure, see: Lin (2008 ▶).
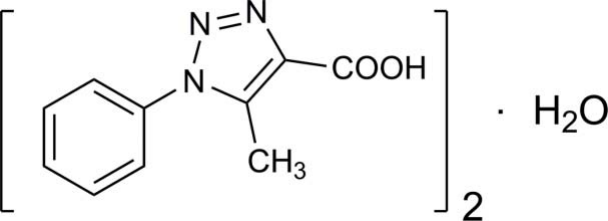


## Experimental

### 

#### Crystal data

2C_10_H_9_N_3_O_2_·H_2_O
*M* *_r_* = 424.42
Monoclinic, 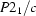

*a* = 6.7419 (13) Å
*b* = 15.842 (3) Å
*c* = 19.643 (4) Å
β = 99.82 (3)°
*V* = 2067.2 (7) Å^3^
*Z* = 4
Mo *K*α radiation
μ = 0.10 mm^−1^
*T* = 293 K
0.42 × 0.38 × 0.35 mm

#### Data collection

Rigaku SCXmini diffractometer
Absorption correction: multi-scan (*CrystalClear*; Rigaku, 2005 ▶) *T*_min_ = 0.947, *T*_max_ = 0.951
20838 measured reflections
4733 independent reflections
3206 reflections with *I* > 2σ(*I*)
*R*_int_ = 0.056

#### Refinement

*R*[*F* ^2^ > 2σ(*F* ^2^)] = 0.052
*wR*(*F* ^2^) = 0.172
*S* = 0.87
4733 reflections
292 parameters
H atoms treated by a mixture of independent and constrained refinement
Δρ_max_ = 0.21 e Å^−3^
Δρ_min_ = −0.19 e Å^−3^

### 

Data collection: *CrystalClear* (Rigaku, 2005 ▶); cell refinement: *CrystalClear*; data reduction: *CrystalClear*; program(s) used to solve structure: *SHELXS97* (Sheldrick, 2008 ▶); program(s) used to refine structure: *SHELXL97* (Sheldrick, 2008 ▶); molecular graphics: *SHELXTL* (Sheldrick, 2008 ▶); software used to prepare material for publication: *SHELXL97*.

## Supplementary Material

Crystal structure: contains datablocks I, global. DOI: 10.1107/S1600536810026243/jh2161sup1.cif

Structure factors: contains datablocks I. DOI: 10.1107/S1600536810026243/jh2161Isup2.hkl

Additional supplementary materials:  crystallographic information; 3D view; checkCIF report

## Figures and Tables

**Table 1:** Hydrogen-bond geometry (Å, °)

*D*—H⋯*A*	*D*—H	H⋯*A*	*D*⋯*A*	*D*—H⋯*A*
O4—H4⋯N3^i^	0.82	1.89	2.704 (2)	170
O1—H1⋯O5	0.82	1.79	2.599 (2)	168
O5—H5*A*⋯O2^ii^	0.85 (4)	2.07 (4)	2.914 (3)	171 (3)
O5—H5*B*⋯N6^iii^	0.83 (4)	2.20 (4)	3.015 (3)	171 (4)

Symmetry codes: (i) 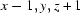; (ii) 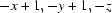; (iii) 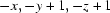.

**Fig. 1.**
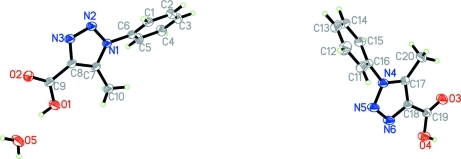
The molecular structure of the title compound, showing the atomic numbering scheme. Displacement ellipsoids are drawn at the 30% probability level.

**Fig. 2.**
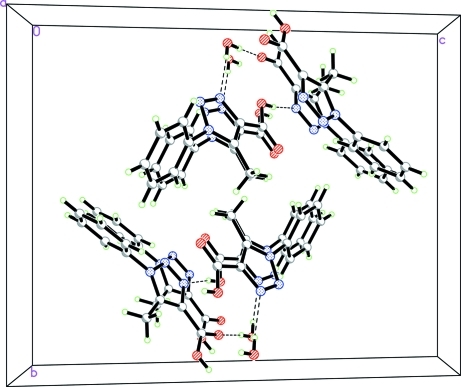
A view of the packing of the title compound, stacking along the a axis.

## supplementary crystallographic information

### Comment

Many triazole-related molecules have received much attention because of their biological activities (Olesen *et al.*, 2003; Tian *et al.*, 2005). Most non-hydrogen atoms of the triazole Ring were coplanar, with the mean deviation from plane of 0.363 (142)° and C11—C16—N4—N5 torsion angle of 57.202 (259)°. The weak π-π packing interactions of the triazole and phenyl ring planes with *Cg*—*Cg* distances from 4.2017 Å to 5.0920 Å (*Cg* is the centroid of the triazole or the phenyl ring planes) stabilized the crystal structure. Because of changes in the external environment, the title compound crystallize in different space group (Lin *et al.*, 2008) and there is a water molecular in the asymmetric unit which transform the method of connection of hydrogen bonds.

As a continuation of our study of phase transition materials, including organicligands (Li *et al.*, 2008), metal-organic coordination compounds (Zhang *et al.*, 2009),the dielectric constant of 5-methyl-1-phenyl-1*H*-1,2,3-triazole-4-carboxylic acid hydrate compound as a function of temperature indicates that the permittivity is basically temperature-independent (dielectric constant equaling to 5.2 to 8.2), suggesting that there may be no distinct phase transition occurred within the measured temperature range.

### Experimental

The title compound was prepared from azidobenzene according to the reported method (El Khadem *et al.*, 1968). The colourless prisms (average size: 0.8×0.8×2.0 mm) were obtained by slow evaporation from ethanol solution at room temperature for 3 days.

### Refinement

All the H atoms attached to the carbon atoms were constrained in a riding motion approximation. Caryl—H=0.93 Å, with *U*_iso_(H)=1.2Ueq(C). Cmethyl—H=0.96 Å, with *U*_iso_(H)=1.5Ueq(C). The hydroxyl hydrogen was refined freely.

### Crystal data

**Table:**

2C_10_H_9_N_3_O_2_·H_2_O	*F*(000) = 888
*M**_r_* = 424.42	*D*_x_ = 1.364 Mg m^−^^3^
Monoclinic, *P*2_1_/*c*	Mo *K*α radiation, λ = 0.71073 Å
Hall symbol: -P 2ybc	Cell parameters from 8429 reflections
*a* = 6.7419 (13) Å	θ = 3.1–27.7°
*b* = 15.842 (3) Å	µ = 0.10 mm^−^^1^
*c* = 19.643 (4) Å	*T* = 293 K
β = 99.82 (3)°	Prism, colorless
*V* = 2067.2 (7) Å^3^	0.42 × 0.38 × 0.35 mm
*Z* = 4	

### Data collection

**Table:**

Rigaku SCXmini diffractometer	4733 independent reflections
Radiation source: fine-focus sealed tube	3206 reflections with *I* > 2σ(*I*)
graphite	*R*_int_ = 0.056
Detector resolution: 13.6612 pixels mm^-1^	θ_max_ = 27.5°, θ_min_ = 3.1°
CCD_Profile_fitting scans	*h* = −8→8
Absorption correction: multi-scan (*CrystalClear*; Rigaku, 2005)	*k* = −20→20
*T*_min_ = 0.947, *T*_max_ = 0.951	*l* = −25→25
20838 measured reflections	

### Refinement

**Table:**

Refinement on *F*^2^	Primary atom site location: structure-invariant direct methods
Least-squares matrix: full	Secondary atom site location: difference Fourier map
*R*[*F*^2^ > 2σ(*F*^2^)] = 0.052	Hydrogen site location: inferred from neighbouring sites
*wR*(*F*^2^) = 0.172	H atoms treated by a mixture of independent and constrained refinement
*S* = 0.87	*w* = 1/[σ^2^(*F*_o_^2^) + (0.1083*P*)^2^ + 0.6293*P*] where *P* = (*F*_o_^2^ + 2*F*_c_^2^)/3
4733 reflections	(Δ/σ)_max_ < 0.001
292 parameters	Δρ_max_ = 0.21 e Å^−^^3^
0 restraints	Δρ_min_ = −0.19 e Å^−^^3^

### Special details

**Table:**

Geometry. All e.s.d.'s (except the e.s.d. in the dihedral angle between two l.s. planes) are estimated using the full covariance matrix. The cell e.s.d.'s are taken into account individually in the estimation of e.s.d.'s in distances, angles and torsion angles; correlations between e.s.d.'s in cell parameters are only used when they are defined by crystal symmetry. An approximate (isotropic) treatment of cell e.s.d.'s is used for estimating e.s.d.'s involving l.s. planes.
Refinement. Refinement of *F*^2^ against ALL reflections. The weighted *R*-factor *wR* and goodness of fit *S* are based on *F*^2^, conventional *R*-factors *R* are based on *F*, with *F* set to zero for negative *F*^2^. The threshold expression of *F*^2^ > σ(*F*^2^) is used only for calculating *R*-factors(gt) *etc*. and is not relevant to the choice of reflections for refinement. *R*-factors based on *F*^2^ are statistically about twice as large as those based on *F*, and *R*- factors based on ALL data will be even larger.

### Fractional atomic coordinates and isotropic or equivalent isotropic displacement parameters (Å^2^)

**Table:**

	*x*	*y*	*z*	*U*_iso_*/*U*_eq_	
O4	−0.2245 (3)	0.25435 (10)	1.04831 (9)	0.0616 (5)	
H4	−0.3031	0.2478	1.0754	0.092*	
N1	0.3101 (2)	0.21148 (10)	0.20357 (8)	0.0427 (4)	
O1	0.2105 (2)	0.44007 (10)	0.09705 (9)	0.0604 (5)	
H1	0.2133	0.4802	0.0709	0.091*	
O2	0.4704 (3)	0.39675 (10)	0.05055 (9)	0.0658 (5)	
N3	0.4944 (3)	0.25050 (10)	0.13134 (10)	0.0505 (5)	
O3	−0.1197 (3)	0.12701 (11)	1.08533 (9)	0.0725 (5)	
N6	0.0253 (3)	0.25962 (11)	0.95482 (10)	0.0515 (5)	
N4	0.2420 (2)	0.17006 (10)	0.93228 (9)	0.0426 (4)	
C17	0.1698 (3)	0.13594 (13)	0.98605 (10)	0.0416 (5)	
N5	0.1530 (3)	0.24561 (11)	0.91340 (10)	0.0542 (5)	
C16	0.3809 (3)	0.13618 (13)	0.89167 (10)	0.0427 (5)	
C7	0.2327 (3)	0.28716 (12)	0.18016 (10)	0.0387 (4)	
C9	0.3516 (3)	0.38637 (12)	0.08889 (11)	0.0438 (5)	
C8	0.3532 (3)	0.31110 (12)	0.13320 (10)	0.0407 (4)	
C6	0.2526 (3)	0.15587 (12)	0.25486 (10)	0.0406 (4)	
C18	0.0303 (3)	0.19342 (12)	0.99957 (10)	0.0397 (4)	
C19	−0.1097 (3)	0.18755 (13)	1.04937 (10)	0.0435 (5)	
N2	0.4702 (3)	0.18997 (11)	0.17417 (10)	0.0544 (5)	
C4	0.0066 (4)	0.07727 (15)	0.30104 (13)	0.0555 (6)	
H4A	−0.1262	0.0602	0.2992	0.067*	
C3	0.1503 (4)	0.04942 (14)	0.35351 (12)	0.0539 (6)	
H3	0.1157	0.0135	0.3871	0.065*	
C1	0.3999 (3)	0.12818 (13)	0.30735 (11)	0.0468 (5)	
H1A	0.5329	0.1452	0.3096	0.056*	
C11	0.5459 (3)	0.18340 (15)	0.88273 (12)	0.0535 (5)	
H11	0.5721	0.2350	0.9051	0.064*	
C5	0.0549 (3)	0.13048 (14)	0.25048 (11)	0.0495 (5)	
H5	−0.0435	0.1487	0.2144	0.059*	
C10	0.0630 (3)	0.32936 (14)	0.20576 (12)	0.0515 (5)	
H10A	−0.0615	0.3140	0.1770	0.077*	
H10B	0.0802	0.3895	0.2044	0.077*	
H10C	0.0606	0.3119	0.2524	0.077*	
C15	0.3431 (4)	0.05856 (14)	0.86021 (12)	0.0542 (6)	
H15	0.2322	0.0268	0.8671	0.065*	
C2	0.3460 (4)	0.07454 (14)	0.35661 (12)	0.0528 (5)	
H2	0.4441	0.0552	0.3924	0.063*	
C13	0.6347 (4)	0.0763 (2)	0.80763 (13)	0.0709 (8)	
H13	0.7194	0.0565	0.7784	0.085*	
C20	0.2496 (4)	0.05710 (17)	1.02157 (13)	0.0694 (8)	
H20A	0.2171	0.0100	0.9909	0.104*	
H20B	0.1901	0.0490	1.0621	0.104*	
H20C	0.3931	0.0614	1.0346	0.104*	
C12	0.6716 (4)	0.1527 (2)	0.83991 (14)	0.0705 (7)	
H12	0.7827	0.1843	0.8329	0.085*	
C14	0.4724 (4)	0.02880 (17)	0.81842 (12)	0.0644 (7)	
H14	0.4496	−0.0237	0.7974	0.077*	
O5	0.2055 (3)	0.58080 (13)	0.02879 (12)	0.0749 (6)	
H5A	0.290 (5)	0.591 (2)	0.0025 (18)	0.098 (11)*	
H5B	0.131 (6)	0.622 (3)	0.0310 (19)	0.111 (13)*	

### Atomic displacement parameters (Å^2^)

**Table:**

	*U*^11^	*U*^22^	*U*^33^	*U*^12^	*U*^13^	*U*^23^
O4	0.0721 (11)	0.0477 (9)	0.0777 (11)	0.0177 (8)	0.0487 (9)	0.0112 (8)
N1	0.0477 (9)	0.0344 (9)	0.0519 (10)	0.0046 (7)	0.0252 (8)	0.0031 (7)
O1	0.0609 (10)	0.0480 (9)	0.0805 (12)	0.0167 (8)	0.0356 (9)	0.0221 (8)
O2	0.0814 (12)	0.0502 (9)	0.0792 (11)	0.0107 (8)	0.0519 (10)	0.0132 (8)
N3	0.0571 (11)	0.0373 (9)	0.0654 (12)	0.0075 (8)	0.0342 (9)	0.0056 (8)
O3	0.0995 (14)	0.0625 (11)	0.0671 (11)	0.0289 (10)	0.0472 (10)	0.0255 (9)
N6	0.0569 (11)	0.0385 (9)	0.0674 (12)	0.0102 (8)	0.0337 (9)	0.0083 (8)
N4	0.0434 (9)	0.0380 (9)	0.0496 (10)	0.0080 (7)	0.0168 (7)	0.0025 (7)
C17	0.0434 (10)	0.0429 (11)	0.0397 (10)	0.0091 (8)	0.0107 (8)	0.0019 (8)
N5	0.0615 (11)	0.0382 (10)	0.0722 (13)	0.0129 (8)	0.0377 (10)	0.0119 (8)
C16	0.0426 (10)	0.0445 (11)	0.0434 (10)	0.0126 (9)	0.0143 (8)	0.0035 (8)
C7	0.0409 (10)	0.0353 (10)	0.0428 (10)	0.0008 (8)	0.0154 (8)	−0.0040 (8)
C9	0.0457 (11)	0.0377 (10)	0.0518 (12)	0.0008 (9)	0.0193 (9)	−0.0020 (8)
C8	0.0440 (10)	0.0341 (10)	0.0482 (11)	0.0017 (8)	0.0194 (9)	−0.0038 (8)
C6	0.0492 (11)	0.0319 (9)	0.0452 (11)	0.0011 (8)	0.0209 (9)	−0.0006 (8)
C18	0.0414 (10)	0.0377 (10)	0.0415 (10)	0.0045 (8)	0.0115 (8)	0.0006 (8)
C19	0.0514 (11)	0.0403 (11)	0.0414 (11)	0.0072 (9)	0.0155 (9)	0.0002 (8)
N2	0.0628 (12)	0.0400 (10)	0.0702 (12)	0.0116 (8)	0.0394 (10)	0.0097 (8)
C4	0.0495 (12)	0.0546 (13)	0.0659 (15)	−0.0092 (10)	0.0195 (11)	0.0070 (11)
C3	0.0665 (14)	0.0445 (12)	0.0555 (13)	−0.0007 (10)	0.0238 (11)	0.0096 (10)
C1	0.0434 (11)	0.0413 (11)	0.0586 (13)	0.0047 (9)	0.0169 (9)	−0.0021 (9)
C11	0.0489 (12)	0.0584 (13)	0.0560 (13)	0.0021 (10)	0.0169 (10)	−0.0043 (10)
C5	0.0487 (12)	0.0498 (12)	0.0508 (12)	−0.0049 (9)	0.0106 (9)	0.0055 (10)
C10	0.0534 (12)	0.0492 (12)	0.0579 (13)	0.0065 (10)	0.0271 (10)	−0.0006 (10)
C15	0.0613 (13)	0.0449 (12)	0.0584 (14)	0.0085 (10)	0.0157 (11)	−0.0012 (10)
C2	0.0604 (13)	0.0459 (12)	0.0528 (13)	0.0092 (10)	0.0120 (10)	0.0075 (10)
C13	0.0699 (17)	0.090 (2)	0.0589 (15)	0.0279 (15)	0.0283 (13)	−0.0044 (14)
C20	0.0859 (18)	0.0688 (16)	0.0585 (15)	0.0407 (14)	0.0265 (13)	0.0226 (12)
C12	0.0535 (14)	0.091 (2)	0.0744 (17)	0.0013 (13)	0.0306 (12)	−0.0034 (15)
C14	0.0805 (17)	0.0569 (14)	0.0582 (14)	0.0247 (13)	0.0186 (12)	−0.0081 (11)
O5	0.0685 (12)	0.0576 (11)	0.1075 (16)	0.0208 (10)	0.0409 (11)	0.0379 (10)

### Geometric parameters (Å, °)

**Table:**

O4—C19	1.309 (2)	C4—C3	1.362 (3)
O4—H4	0.8200	C4—C5	1.383 (3)
N1—N2	1.352 (2)	C4—H4A	0.9300
N1—C7	1.356 (3)	C3—C2	1.370 (3)
N1—C6	1.440 (2)	C3—H3	0.9300
O1—C9	1.306 (2)	C1—C2	1.382 (3)
O1—H1	0.8200	C1—H1A	0.9300
O2—C9	1.201 (2)	C11—C12	1.381 (3)
N3—N2	1.304 (2)	C11—H11	0.9300
N3—C8	1.357 (2)	C5—H5	0.9300
O3—C19	1.200 (2)	C10—H10A	0.9600
N6—N5	1.300 (2)	C10—H10B	0.9600
N6—C18	1.365 (3)	C10—H10C	0.9600
N4—C17	1.349 (2)	C15—C14	1.378 (3)
N4—N5	1.362 (2)	C15—H15	0.9300
N4—C16	1.434 (2)	C2—H2	0.9300
C17—C18	1.367 (3)	C13—C12	1.370 (4)
C17—C20	1.486 (3)	C13—C14	1.373 (4)
C16—C11	1.376 (3)	C13—H13	0.9300
C16—C15	1.380 (3)	C20—H20A	0.9600
C7—C8	1.382 (3)	C20—H20B	0.9600
C7—C10	1.485 (3)	C20—H20C	0.9600
C9—C8	1.475 (3)	C12—H12	0.9300
C6—C1	1.376 (3)	C14—H14	0.9300
C6—C5	1.381 (3)	O5—H5A	0.85 (4)
C18—C19	1.474 (3)	O5—H5B	0.83 (4)
			
C19—O4—H4	109.5	C4—C3—H3	120.2
N2—N1—C7	111.55 (15)	C2—C3—H3	120.2
N2—N1—C6	118.32 (15)	C6—C1—C2	118.6 (2)
C7—N1—C6	130.07 (16)	C6—C1—H1A	120.7
C9—O1—H1	109.5	C2—C1—H1A	120.7
N2—N3—C8	109.73 (16)	C16—C11—C12	118.7 (2)
N5—N6—C18	109.01 (16)	C16—C11—H11	120.7
C17—N4—N5	111.38 (15)	C12—C11—H11	120.7
C17—N4—C16	129.99 (17)	C6—C5—C4	118.4 (2)
N5—N4—C16	118.46 (16)	C6—C5—H5	120.8
N4—C17—C18	103.72 (17)	C4—C5—H5	120.8
N4—C17—C20	123.68 (18)	C7—C10—H10A	109.5
C18—C17—C20	132.41 (19)	C7—C10—H10B	109.5
N6—N5—N4	106.70 (16)	H10A—C10—H10B	109.5
C11—C16—C15	121.2 (2)	C7—C10—H10C	109.5
C11—C16—N4	119.10 (19)	H10A—C10—H10C	109.5
C15—C16—N4	119.62 (19)	H10B—C10—H10C	109.5
N1—C7—C8	103.45 (16)	C14—C15—C16	119.0 (2)
N1—C7—C10	123.88 (17)	C14—C15—H15	120.5
C8—C7—C10	132.61 (18)	C16—C15—H15	120.5
O2—C9—O1	124.44 (19)	C3—C2—C1	120.9 (2)
O2—C9—C8	122.83 (18)	C3—C2—H2	119.6
O1—C9—C8	112.72 (17)	C1—C2—H2	119.6
N3—C8—C7	108.59 (17)	C12—C13—C14	120.0 (2)
N3—C8—C9	119.39 (17)	C12—C13—H13	120.0
C7—C8—C9	132.02 (17)	C14—C13—H13	120.0
C1—C6—C5	121.31 (19)	C17—C20—H20A	109.5
C1—C6—N1	118.25 (18)	C17—C20—H20B	109.5
C5—C6—N1	120.44 (19)	H20A—C20—H20B	109.5
N6—C18—C17	109.19 (17)	C17—C20—H20C	109.5
N6—C18—C19	121.87 (17)	H20A—C20—H20C	109.5
C17—C18—C19	128.80 (18)	H20B—C20—H20C	109.5
O3—C19—O4	124.27 (19)	C13—C12—C11	120.7 (3)
O3—C19—C18	123.24 (18)	C13—C12—H12	119.6
O4—C19—C18	112.46 (17)	C11—C12—H12	119.6
N3—N2—N1	106.67 (15)	C13—C14—C15	120.4 (2)
C3—C4—C5	121.1 (2)	C13—C14—H14	119.8
C3—C4—H4A	119.4	C15—C14—H14	119.8
C5—C4—H4A	119.4	H5A—O5—H5B	112 (3)
C4—C3—C2	119.7 (2)		

### Hydrogen-bond geometry (Å, °)

**Table:**

*D*—H···*A*	*D*—H	H···*A*	*D*···*A*	*D*—H···*A*
O4—H4···N3^i^	0.82	1.89	2.704 (2)	170
O1—H1···O5	0.82	1.79	2.599 (2)	168
O5—H5A···O2^ii^	0.85 (4)	2.07 (4)	2.914 (3)	171 (3)
O5—H5B···N6^iii^	0.83 (4)	2.20 (4)	3.015 (3)	171 (4)

Symmetry codes: (i) *x*−1, *y*, *z*+1; (ii) −*x*+1, −*y*+1, −*z*; (iii) −*x*, −*y*+1, −*z*+1.
